# Does hypoxemia aggravate sepsis-associated acute kidney injury? Integrated clinical and experimental evidence

**DOI:** 10.1186/s40635-025-00840-x

**Published:** 2025-12-21

**Authors:** Haoyun Mao, Jiayue Xu, Yueniu Zhu, Xiangmei Kong, Jiru Li, Xiaodong Zhu, Yaya Xu

**Affiliations:** Department of Pediatric Critical Care Medicine, Xinhua Hospital, Affiliated to the Medical School of Shanghai Jiaotong University, 1665 Kongjiang Road, Yangpu District, Shanghai, 200092 China

**Keywords:** Acute kidney injury, Hypoxia, Mitochondrial dysfunction, Sepsis

## Abstract

**Background:**

Sepsis-associated acute kidney injury (SA-AKI) is a common and severe complication in critically ill patients, but the association between hypoxemia and renal dysfunction remains uncertain.

**Method:**

We retrospectively analyzed 2292 patients with SA-AKI from the MIMIC-IV database and stratified them into four groups based on their highest arterial partial pressure of oxygen (PO₂) within 24 h of admission: < 60 mmHg, ≥ 60 to < 80 mmHg, ≥ 80 to  < 100 mmHg, and ≥ 100 mmHg. Associations between PO₂ and renal injury markers (serum creatinine [SCr] and blood urea nitrogen [BUN]) were evaluated using multivariable regression analyses, and survival outcomes were compared with Kaplan–Meier methods. To explore mechanistic pathways, a murine model was established with four experimental conditions: normoxia, hypoxia (10% O₂), lipopolysaccharide (LPS)-induced sepsis, and combined sepsis plus hypoxia. Serum biochemical parameters, histological injury, and protein expression of hypoxia-inducible factor-1α (HIF-1α) were measured at 6, 24, and 48 h. Mitochondrial autophagy was assessed by LC3 and TOMM20 immunofluorescence colocalization.

**Result:**

Patients with lower PO₂ had higher illness severity and unadjusted BUN and SCr levels, multivariable analyses revealed no independent association between PO₂ and renal injury markers. Survival differed significantly across groups, with the ≥ 100 mmHg group showing the best outcomes (log-rank P < 0.001). In animal experiments, sepsis groups developed increased SCr and BUN at 24 and 48 h, but combined hypoxia did not exacerbate these parameters compared to sepsis alone. Histological analysis revealed severe tubular injury with no significant aggravation in the sepsis-plus hypoxia group. HIF-1α expression was lowest in sepsis-only kidneys but markedly upregulated in the sepsis-plus-hypoxia group at 6 h. Immunofluorescence demonstrated less colocalization of LC3 and TOMM20 in the sepsis-only group than in sepsis-plus-hypoxia mice, suggesting more efficient mitophagy with hypoxemia.

**Conclusions:**

These clinical and experimental findings indicate that hypoxemia was not independently associated with aggravated renal injury in SA-AKI and may activate HIF-1α and promote adaptive mitophagy. This challenges the conventionally held belief that hypoxemia is uniformly detrimental to renal function during sepsis.

**Supplementary Information:**

The online version contains supplementary material available at 10.1186/s40635-025-00840-x.

## Background

Sepsis is the most common cause of acute kidney injury (AKI) in critically ill patients [1]. Existing studies indicate that over one-third of sepsis patients develop AKI within the first seven days of their intensive care unit (ICU) stay, which is associated with higher mortality rates [2]. Multiple mechanisms can contribute to injury in sepsis-associated AKI (SA-AKI), among which renal hypoxia might play a pivotal role. The kidney is inherently susceptible to oxygen deficiency due to its complex architecture, heterogeneous perfusion, and high metabolic demand. Hypoxia not only predominates during the acute phase of injury but also contributes to the progression from AKI to chronic kidney disease [3]. Some evidence suggests that hypoxia may aggravate kidney injury by inducing mitochondrial dysfunction in tubular epithelial cells, leading to impaired energy metabolism and apoptosis [4, 5]. Therefore, some advocate proactive oxygen therapy in SA‑AKI. In a lipopolysaccharide (LPS)-induced endotoxemia rat model, a single early session of hyperbaric oxygen therapy significantly reduced renal tubular apoptosis by suppressing p53 activation and cytochrome C release, demonstrating a renoprotective effect [6]. Clinically, Kim et al. observed that early invasive mechanical ventilation—initiated on the first day of ICU admission—was associated with significantly better outcomes in septic patients compared to later initiation [7].

However, others suggest that maintaining moderate hypoxia may not worsen injury and could activate renal self-repair mechanisms. Our previous work demonstrated that rats exposed to mild hypoxia (10% O₂) for two weeks exhibited marked upregulation of hypoxia-inducible factor-1α (HIF‑1α) and vascular endothelial growth factor (VEGF), increased microvascular density, and improved renal injury [8]. Importantly, overly aggressive oxygenation and interventions such as mechanical ventilation or extracorporeal membrane oxygenation can trigger oxidative stress and tissue injury, potentially disrupting repair pathways [9, 10]. Animal data further indicate that hyperoxia induces systemic inflammation, exacerbating organ damage [11]. Clinically, Vemuri et al. found that nearly 39% of patients undergoing invasive mechanical ventilation developed AKI—significantly more than those not ventilated [12].

This study investigates the association between hypoxemia and renal dysfunction in both septic patients and an LPS-induced sepsis mouse model. Patients were categorized into four groups according to their highest arterial partial pressure of oxygen (PO₂) within the first 24 h after admission (< 60 mmHg, ≥ 60– < 80 mmHg, ≥ 80– < 100 mmHg, and ≥ 100 mmHg) to assess renal function differences. In parallel, renal pathology was compared under normoxic and hypoxic conditions in septic mice. Mitochondrial injury was evaluated to elucidate the mechanisms by which hypoxia influences SA-AKI.

## Results

### Hypoxemia is not independently associated with kidney injury severity in SA-AKI patients

Table 1 summarizes the baseline characteristics of the four PO₂ groups. Patients with lower PO₂ levels (< 60 mmHg) had higher Sequential Organ Failure Assessment (SOFA), Acute Physiology Score III (APS-III), and Charlson Comorbidity Index scores than those with higher PO₂ (all P < 0.05), indicating greater illness severity in hypoxemic patients. Arterial oxygen levels showed clear stratification among the four groups (H = 1500.9, P < 0.001). Lactate concentrations were slightly higher in the ≥ 100 mmHg group (2.3 [1.7–3.3] mmol/L) than in the lower PO₂ groups (2.0 [1.3–3.1] mmol/L), reaching statistical significance (H = 31.5, P < 0.001). Inflammatory markers also differed significantly: both white blood cell (WBC) count and neutrophil-to-lymphocyte ratio (NLR) were elevated in patients with lower PO₂ levels, particularly in the PO₂ < 60 mmHg and 60–80 mmHg groups (P = 0.002 and P < 0.001, respectively).

**Table 1:** Comparisons of baseline characteristics (n = 2992)

Variables	< 60 mmHg (n = 132)	≥ 60 and < 80 mmHg (n = 221)	≥ 80 and < 100 mmHg (n = 269)	≥ 100 mmHg (n = 2370)	*H/*χ^2^ value	*P* Value
Age (year, median [IQR])	61.5 (52.0, 72.5)	62.0 (54.0, 72.0)	65.0 (54.0, 75.0)	65.0 (55.0, 74.0)	3.5	0.324
Male (%)	72 (54.5)	121 (54.8)	160 (59.5)	1503 (63.4)^b^	10.792	0.013
Ethnicity, n (%)						
American Indian or Alaska Native	1 (0.8)	2 (0.9)	0 (0.0)	0 (0.0)	25.920	0.086
Asian	5 (3.8)	8 (3.6)	11 (4.1)	58 (2.4)		
Black/African American	10 (7.6)	14 (6.3)	13 (4.8)	148 (6.2)		
Hispanic/Latino	2 (1.5)	6 (2.7)	10 (3.7)	69 (2.9)		
White	89 (67.4)	157 (71.0)	199 (74.0)	1724 (72.7)		
Other	5 (3.8)	13 (5.9)	13 (4.8)	125 (5.3)		
Unkow	20 (15.2)	21 (9.5)	23 (8.6)	246 (10.4)		
First care unit, n (%)						
CVICU	4 (3.0)	8 (3.6)	7 (2.6)	1097 (46.3)	658.858	< 0.001
CCU	5 (3.8)	12 (5.4)	10 (3.7)	54 (2.3)		
MICU	55 (41.7)	59 (26.7)	75 (27.9)	230 (9.7)		
MICU/SICU	41 (31.1)	78 (35.3)	79 (29.4)	279 (11.8)		
Neuro Intermediate	1 (0.8)	2 (0.9)	2 (0.7)	3 (0.1)		
Neuro SICU	5 (3.8)	5 (2.3)	8 (3.0)	24 (1.0)		
SICU	14 (10.6)	32 (14.5)	40 (14.9)	389 (16.4)		
TSICU	7 (5.3)	25 (11.3)	48 (17.8)	294 (12.4)		
Severity of illness (median [IQR])						
SOFA score	6.0 (4.0, 9.0)	5.0 (3.0, 7.0)^a^	6.0 (4.0, 8.0)^b^	5.0 (4.0, 8.0)	12.9	0.005
APS-III score	56.0 (48.0, 56.0)	56.0 (44.0, 56.0)	56.0 (43.0, 56.0)	44.0 (32.0, 56.0)^abc^	134.4	< 0.001
Charlson comorbidity index	5.0 (4.0, 7.0)	5.0 (4.0, 8.0)	5.0 (4.0, 7.0)	5.0 (3.0, 6.0)^bc^	37.4	< 0.001
Oxygen metabolism variables during the first 24 h in ICU (median [IQR])						
PO_2_ (mmHg)	46.0 (38.0, 53.0)	71.0 (66.0, 75.0)	90.0 (85.0, 94.0)^ab^	117.8 (108.0, 131.3)^abc^	1500.9	< 0.001
PCO_2_ (mmHg)	40.0 (36.0, 46.0)	40.0 (34.0, 46.0)	42.0 (36.0, 51.0)^b^	47.0 (41.0, 51.0)^abc^	150.7	< 0.001
Lac (mmol/L)	2.0 (1.5, 3.0)	2.0 (1.3, 3.0)	2.0 (1.4, 3.1)	2.3 (1.7, 3.3)^bc^	31.5	< 0.001
Inflammatory variables during the first 24 h in ICU (median [IQR])						
WBC (× 10^9^/L)	13.9 (9.0, 19.2)	13.2 (9.5, 17.9)	14.1 (9.9, 19.7)	14.8 (11.1, 19.2)^b^	14.3	0.002
NLR	10.1 (5.0, 17.0)	11.4 (6.1, 18.4)	11.1 (5.4, 20.6)	7.0 (4.1, 13.3)^abc^	55.6	< 0.001

Data were analyzed using the Kruskal–Wallis test (H) for continuous variables or the Chi-square test (χ^2^) for categorical variables. Pairwise comparisons between groups were performed when appropriate. Compared with the < 60 mmHg group

*AKI* acute kidney injury, *APS-III* Acute Physiology Score III, *CCU* coronary care unit, *CVICU* cardiovascular intensive care unit, *IQR* interquartile range, *Lac* lactate, *MICU* medical intensive care unit, *MICU/SICU* combined medical–surgical intensive care unit, *NLR* neutrophil-to-lymphocyte ratio, *PCO₂* partial pressure of carbon dioxide, *aO₂* partial pressure of oxygen, *SICU* surgical intensive care unit, *SOFA* Sequential Organ Failure Assessment, *TSICU* trauma surgical intensive care unit, *WBC* white blood cell

^a^*P* indicates *P* < 0.05; compared with the ≥ 60 and < 80 mmHg group

^b^*P* indicates *P* < 0.05; compared with the ≥ 80 and < 100 mmHg group,^c^*P* indicates *P* < 0.05

**Fig. 1:**
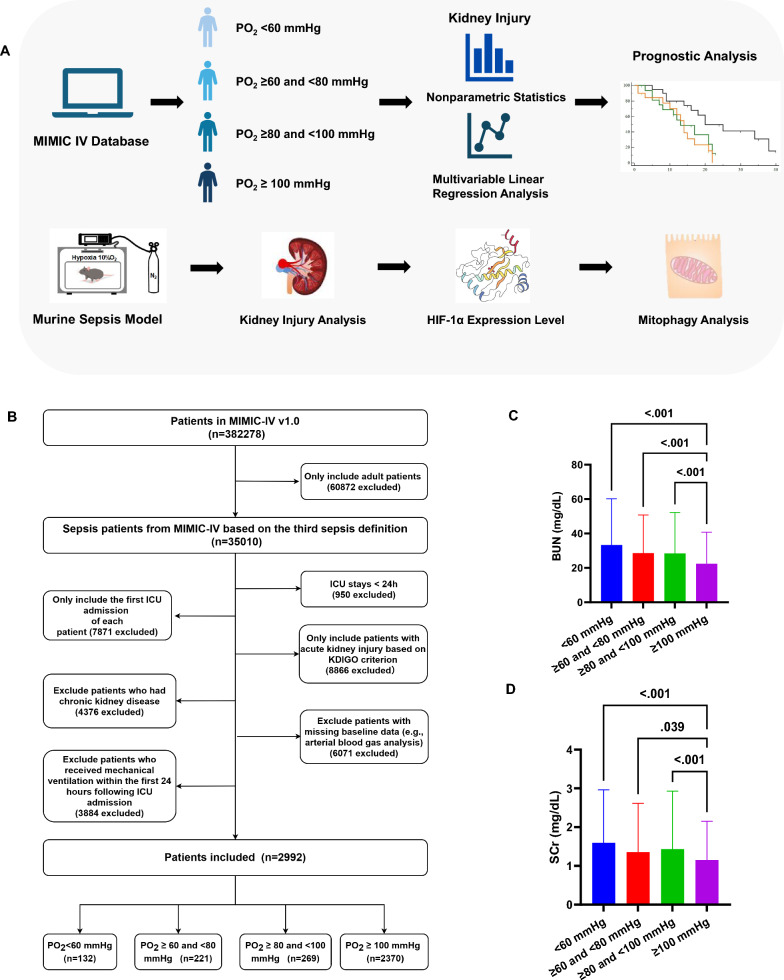
**A** Schematic overview of the study design. Patients with sepsis were identified from the MIMIC-IV database and stratified into four groups based on initial arterial blood gas measurements to evaluate the association between hypoxemia and renal dysfunction. To validate these findings in vivo, an LPS-induced sepsis mouse model was established to compare renal injury under normoxic and hypoxic conditions. Further assessment of mitochondrial function was performed to explore potential underlying mechanisms. **B** Flowchart illustrating the selection of sepsis cases from the MIMIC-IV database. **C** Blood urea nitrogen levels. **D** Serum creatinine levels. *BUN* blood urea nitrogen, *HIF-1α* hypoxia-inducible factor-1 alpha, *ICU* intensive care unit, *KDIGO* Kidney Disease: Improving Global Outcomes, *MIMIC-IV* Medical Information Mart for Intensive Care IV, *PO₂* partial pressure of oxygen, *SCr* serum creatinine

**Fig. 2:**
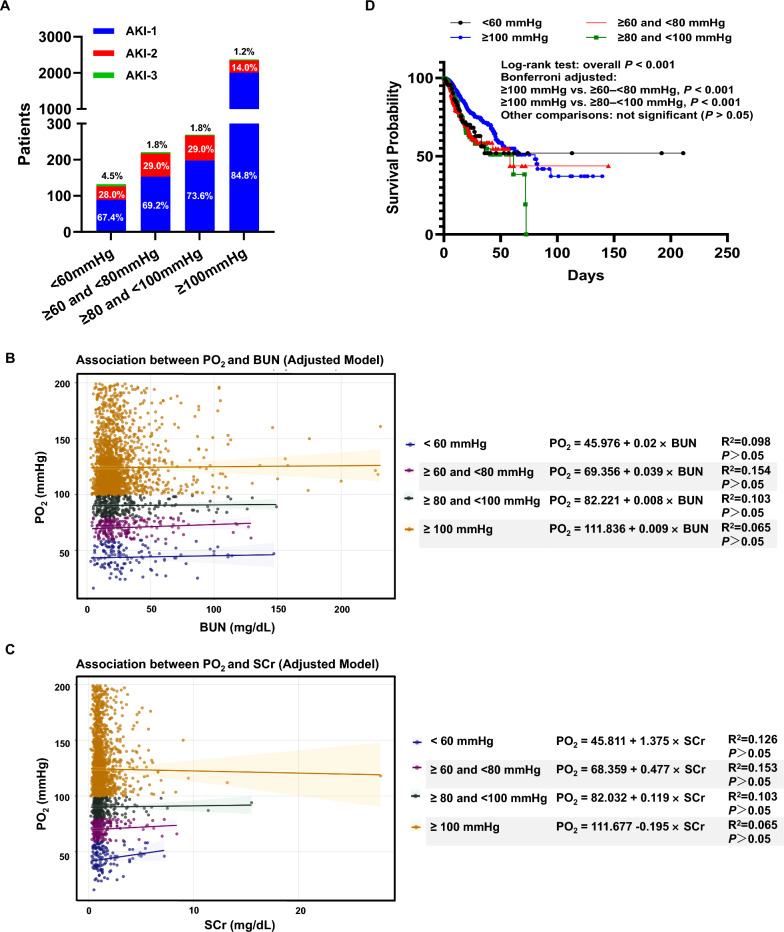
**A** Severity of acute kidney injury based on KDIGO staging criteria (Stage 1, 2, and 3). Association between arterial oxygen tension and BUN (**B**) or SCr (**C**) after multivariable adjustment. Multivariable linear regression models were constructed to evaluate the relationship between PO₂ and BUN or SCr across four oxygenation strata (< 60 mmHg, ≥ 60 to  < 80 mmHg, ≥ 80 to  < 100 mmHg, and ≥ 100 mmHg). Potential confounding factors were controlled for, including age, gender, ethnicity, Sequential Organ Failure Assessment score, Acute Physiology Score III, Charlson Comorbidity Index, white blood cell count, neutrophil-to-lymphocyte ratio, and vasoactive medication use. **D** Adjusted Kaplan–Meier survival curves across four oxygenation groups. Kaplan–Meier analyses were performed to compare survival probabilities among patients with different arterial oxygen tensions (< 60 mmHg, ≥ 60– < 80 mmHg, ≥ 80– < 100 mmHg, and ≥ 100 mmHg). Potential confounding factors were controlled for, including age, gender, ethnicity, Sequential Organ Failure Assessment score, Acute Physiology Score III, Charlson Comorbidity Index, white blood cell count, neutrophil-to-lymphocyte ratio, and vasoactive medication use. *AKI* acute kidney injury, *BUN* blood urea nitrogen, *KDIGO* Kidney Disease: Improving Global Outcomes, *PO₂* partial pressure of oxygen, *SCr* serum creatinine

Figure 1C and Fig. 1D illustrate the distribution of renal injury markers—blood urea nitrogen (BUN) and serum creatinine (SCr) across the four PO₂ groups. Patients in the lower PO₂ categories (< 60 mmHg and 60–80 mmHg) exhibited significantly higher BUN and SCr levels compared with those in the ≥ 80– < 100 mmHg and ≥ 100 mmHg groups (all P < 0.05), indicating association between renal impairment and hypoxemia. We further analyzed the severity distribution of AKI across different oxygenation groups (Fig. 2A). In the PO₂ < 60 mmHg group, AKI-2 and AKI-3 accounted for 28.0% and 4.5% of cases, respectively, whereas these proportions declined to 14.0% and 1.2% in the ≥ 100 mmHg group, indicating that patients with lower arterial oxygen levels tended to develop more severe kidney injury.

Figure 2B and Fig. 2C illustrate the adjusted linear regression analyses between PO₂ and renal injury markers (BUN and SCr) after controlling for potential confounders (including age, gender, ethnicity, SOFA score, APSIII score, Charlson comorbidity index, white blood cell count (WBC), NLR, and vasoactive medication use). No significant associations were observed between PO₂ and either BUN or SCr across the four PO₂ strata (all P > 0.05). The regression slopes were close to zero, and no consistent trend of decreasing BUN or SCr with increasing PO₂ was identified, suggesting that renal injury markers were not independently correlated with PO₂ levels after adjustment for confounding factors.

Figure 2D presents the adjusted Kaplan–Meier (K-M) survival curves for the four PO₂ groups after controlling for potential confounding variables. Overall survival differed significantly among groups (log-rank test, P < 0.001). Patients with higher PO₂ levels (≥ 100 mmHg) demonstrated markedly better survival compared with those in the 60– < 80 mmHg and 80– < 100 mmHg groups (both P < 0.001 after Bonferroni correction), whereas survival differences among the lower PO₂ groups were not statistically significant (P > 0.05).

### Hypoxia does not exacerbate renal injury in a murine sepsis model

**Fig. 3:**
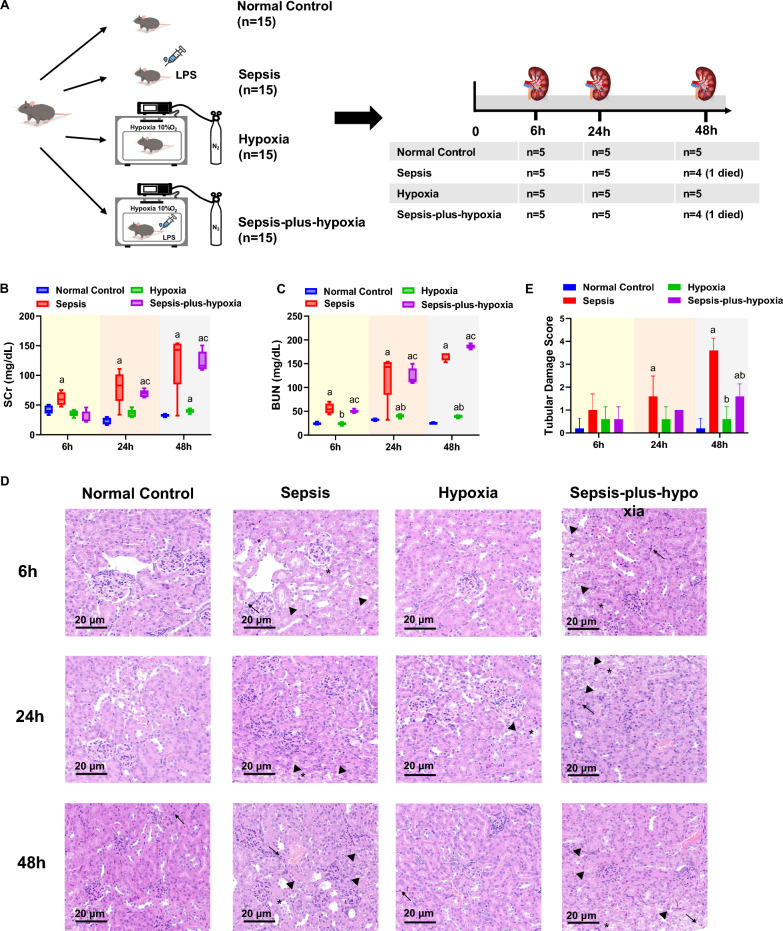
Evaluation of renal injury in an LPS-induced sepsis mouse model under normoxic and hypoxic conditions. **A** Experimental grouping and protocol. Mice were divided into four groups: Normal Control; Sepsis (induced by intraperitoneal injection of LPS, 20 mg/kg; Escherichia coli 055:B5); Hypoxia (exposed to 10% O₂ using the ProOx-100 system); and Sepsis-plus-Hypoxia (septic mice placed in the hypoxic chamber immediately after LPS injection). **B** Serum creatinine levels at 6-, 24-, and 48-h post-induction. **C** Blood urea nitrogen levels at 6, 24, and 48 h post-induction. **D** Representative Kidney histology at 6, 24, and 48 h, with hematoxylin and eosin staining. Magnification × 40. Pathological features include vacuolar degeneration of renal tubular epithelial cells (▲), denudation of the tubular basement membrane with exposed epithelium (*) and lymphocytic infiltration ( →). **E** Tubular damage scores based on H&E-stained sections at 6, 24, and 48 h. Ten random cortical fields per mouse were evaluated. Tubular injury was scored as follows: 0 = no damage; 1 =  < 25%; 2 = 25–50%; 3 = 51–75%; and 4 =  > 75% of tubules showing injury. ^a^P, vs. Normal Control group, P < 0.05; ^b^P, vs. Sepsis group, P < 0.05; ^c^P, vs. Hypoxia group, P < 0.05. *BUN* blood urea nitrogen, *H&E* hematoxylin and eosin, *LPS* lipopolysaccharide, *SCr* serum creatinine

To assess the association between hypoxemia and renal dysfunction in SA-AKI, we established a murine model of sepsis with or without hypoxia exposure (Fig. 3A). At 6 h, arterial blood gas parameters were largely within normal ranges across all groups. By 48 h, PO₂ levels had declined significantly in both the Sepsis and Sepsis-plus-hypoxia groups, with the most pronounced reduction observed in the Sepsis-plus-hypoxia group, reaching 53.0 (39.0, 153.0) mmHg (Table 2). Throughout the experimental period (6 h, 24 h, and 48 h), SCr and BUN levels remained stable in the hypoxia-only group, showing no significant differences from the Normal control group. In contrast, both sepsis-related groups demonstrated substantial elevations in SCr and BUN at 24 h and 48 h, consistent with the development of renal dysfunction. Notably, no significant difference was observed between the Sepsis and Sepsis-plus-hypoxia groups, suggesting that the addition of hypoxia to sepsis may not have further exacerbated renal dysfunction. (Fig. 3B–C).

**Table 2:** Arterial Blood Gas Parameters in Experimental Mouse Groups

Time	Group	*Arterial Blood Gas*		
		PO_2_ (mmHg)	PCO_2_ (mmHg)	SO_2_ (%)
6 h	Normal Control (n = 5)	143.0 (244.6, 135.0)	47.6 ± 0.1	97.5 (96.0, 116.6)
	Sepsis (n = 5)	137.0 (86.0, 194.2)	51.4 ± 6.8	98.0 (98.0, 104.6)
	Hypoxia (n = 5)	124.0 (58.0, 215.3)	66.2 ± 23.8	95.0 (73.0, 122.6)
	Sepsis‑plus‑hypoxia (n = 5)	95.5 (77.1, 123.0)	38.6 ± 4.1	88.5 (79.2, 97.0)
	H/F Value	3.048	6.714	3.747
	P Value	0.384	0.082	0.290
24 h	Normal Control (n = 5)	140.0 (113.0, 189.5)	52.6 ± 10.0	94.0 (98.5, 106.7)
	Sepsis (n = 5)	143.0 (41.0 167.8)	47.1 ± 6.6	90.0 (89.4, 99.0)
	Hypoxia (n = 5)	146.0 (126.0, 175.7)	53.9 ± 11.3	97.0 (97.0, 101.3)
	Sepsis‑plus‑hypoxia (n = 5)	82.3 (60.0, 164.5)	43.0 ± 16.6^ac^	79.0 (69.6, 99.0)
	H/F Value	5.135	8.596	7.622
	P Value	0.162	0.035	0.055
48 h	Normal Control (n = 5)	126.0 (125.0, 138.7)	54.3 ± 2.8	98.0 (98.0, 107.1)
	Sepsis (n = 4)	97.0 (35.0, 170.9)	40.3 ± 4.4	80.0 (79.0, 99.5)
	Hypoxia (n = 5)	123.5 (78.0, 173.4)	55.1 ± 8.9	95.0 (80.0, 105.2)
	Sepsis‑plus‑hypoxia (n = 4)	53.0 (39.0, 153.0)	32.7 ± 2.0	78.5 (78.0, 104.9)
	H/F Value	6.205	2.769	3.990
	P Value	0.102	0.429	0.263

Data are expressed as median (P25, P75) for non-normally distributed variables or mean ± SD for normally distributed variables. Continuous variables with non-normal distribution were analyzed using the Kruskal–Wallis test (H), and those with normal distribution were analyzed using one-way ANOVA (F). Pairwise comparisons between groups were performed when appropriate

PCO₂ arterial partial pressure of carbon dioxide, PO₂ arterial partial pressure of oxygen, SO₂ arterial oxygen saturation

Compared with the Normal Control group, ^a^P < 0.05; compared with the Hypoxia group, ^c^P < 0.05

Histological analysis (Fig. 3D) revealed marked tubular injury in the Sepsis group, characterized by tubular vacuolar degeneration, inflammatory cell infiltration, and denudation of the tubular basement membrane. Similar but less pronounced changes were observed in the Sepsis‑plus‑hypoxia group, while the Hypoxia group exhibited only mild inflammatory cell infiltration. Tubular damage score showed that the Sepsis group had the most severe injury, followed by the Sepsis‑plus‑hypoxia group (Fig. 3E). Figure S1 illustrate the extent of renal tubular injury distribution within the kidney in the Sepsis and Sepsis‑plus‑hypoxia groups at 6, 24, and 48 h.

### Mechanisms underlying the non-aggravating effect of hypoxia on renal injury

**Fig. 4:**
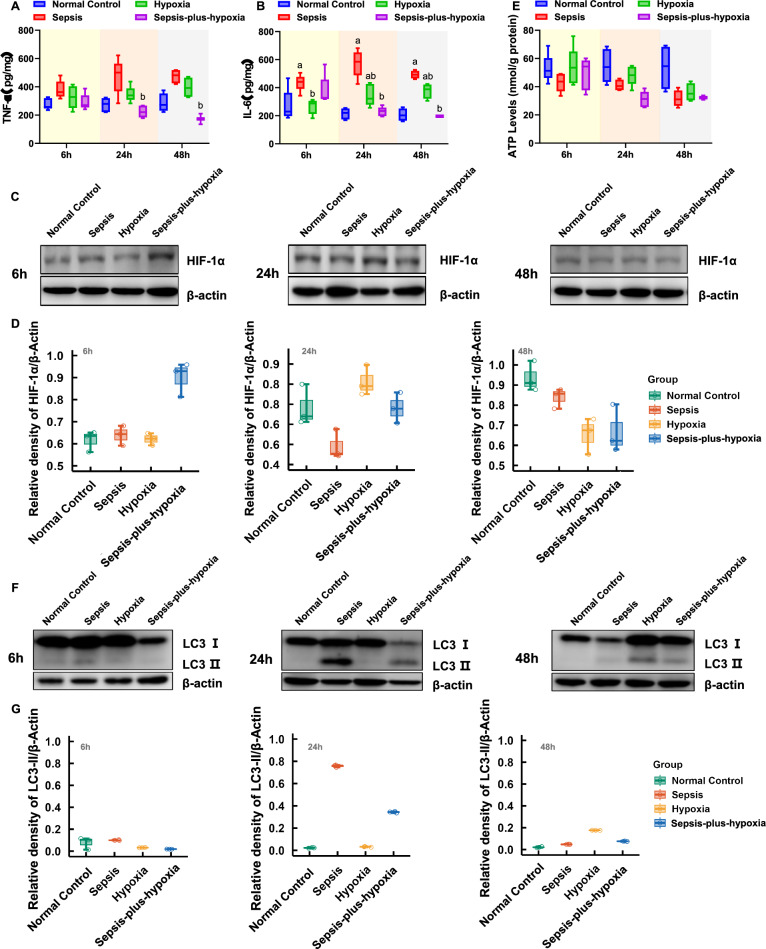
Potential mechanisms of hypoxia-induced renal injury in sepsis. **A** Comparison of serum TNF-α levels at 6, 24, and 48 h among groups. **B** Comparison of serum IL-6 levels at 6, 24, and 48 h among groups. **C** Western blot analysis of renal HIF-1α expression at 6, 24, and 48 h. **D** Densitometric quantification of HIF-1α expression. **E** Renal ATP levels at 6, 24, and 48 h. **F** Western blot analysis of LC3-I and LC3-II expression in kidney tissues at 6, 24, and 48 h. **G** Densitometric quantification of LC3-I and LC3-II expression. Mice were divided into four groups: Normal Control; Sepsis (induced by intraperitoneal injection of LPS, 20 mg/kg; Escherichia coli 055:B5); Hypoxia (exposed to 10% O₂ using the ProOx-100 system); and Sepsis-plus-Hypoxia (septic mice placed in the hypoxic chamber immediately after LPS injection). ^a^P, vs. Normal Control group, P < 0.05; ^b^P, vs. Sepsis group, P < 0.05; When no symbols are shown, the differences among groups are not statistically significant (P > 0.05). *ATP* adenosine triphosphate, *HIF-1α* hypoxia-inducible factor 1 alpha, *IL-6* interleukin-6, *LC3* microtubule-associated protein 1 light chain 3, *TNF-α* tumor necrosis factor alpha, *WB* western blot

To further investigate why hypoxia did not aggravate renal injury in septic conditions—as observed in both clinical and animal models—we explored potential underlying mechanisms (Fig. 4). We first examined the inflammatory response across groups. Surprisingly, the combination of hypoxia and sepsis did not amplify inflammation. Instead, proinflammatory cytokines, tumor necrosis factor-alpha and interleukin-6, were significantly lower in the Sepsis-plus-hypoxia group compared with the Sepsis group at multiple timepoints (Fig. 4A–B). To evaluate renal hypoxic responses, we assessed the relative expression of HIF-1α by western blot (WB). At 6 h, HIF-1α levels were relatively higher in the Sepsis-plus-hypoxia group compared to other groups, whereas the Sepsis group showed the lowest expression. At later time points, HIF-1α expression was relatively higher in the Hypoxia group, while both Sepsis and Sepsis-plus-hypoxia groups showed lower levels, suggesting that although concurrent hypoxia may initially enhance HIF-1α expression under septic conditions, this response is not sustained over time (Fig. 4C–D).

### Investigating mitochondrial metabolism as a potential mediator of kidney injury in response to hypoxia during sepsis

To further elucidate why hypoxia did not aggravate renal injury in septic mice, we evaluated mitochondrial function. we assessed mitochondrial function and autophagy in a murine model. Over the 6‐, 24‐, and 48‐hour post‐induction time points, renal adenosine triphosphate (ATP) levels showed a decreasing trend across the Hypoxia, Sepsis, and Sepsis-plus-hypoxia groups. However, statistical analysis revealed no significant differences in these downward changes among the group (Fig. 4E).

During autophagy, microtubule-associated protein 1 light chain 3 (LC3)-I is lipidated to form LC3-II, which localizes to autophagosomal membranes and serves as a widely accepted marker of autophagic activity [13]. Therefore, we compared the expression levels of LC3-I and LC3-II across groups to assess autophagy status (Fig. 4F–G).

The LC3-II/LC3-I ratio, detected by WB, showed no significant differences among groups at 6 h, indicating minimal autophagic activity (Figure S2). At 24 h, the Sepsis and Sepsis-plus-hypoxia groups exhibited relatively higher ratios, suggesting increased autophagy. At 48 h, the Sepsis group showed a relatively higher LC3-II/LC3-I ratio compared to the Sepsis-plus-hypoxia group, which may indicate altered autophagic activity.

**Fig. 5:**
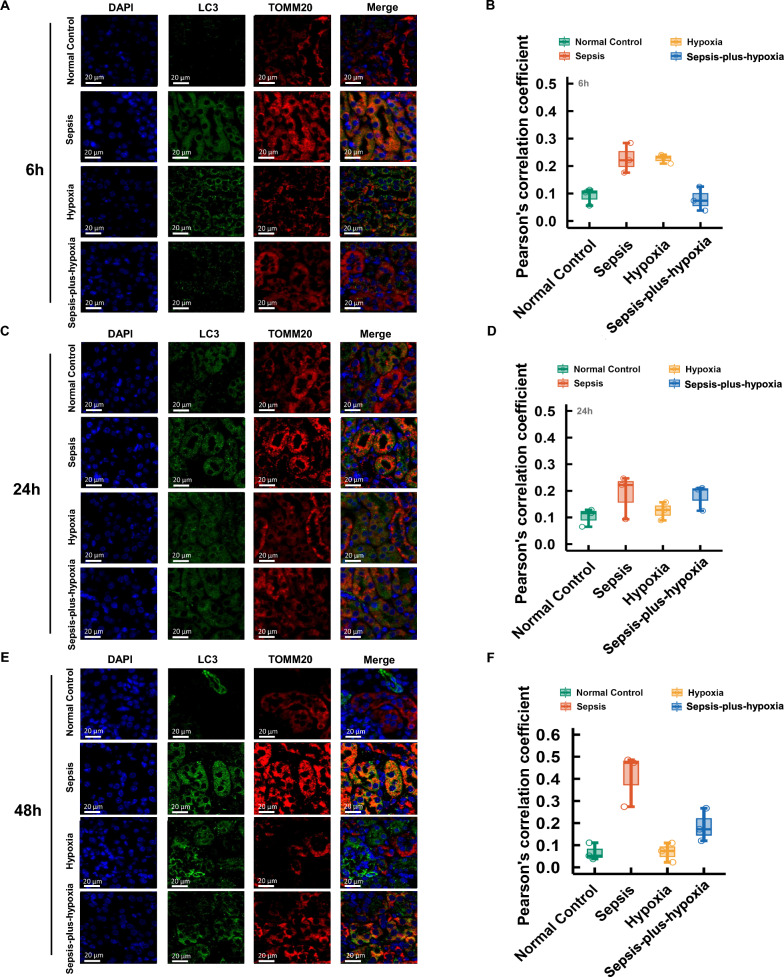
Immunofluorescence staining of TOMM20 (red) and LC3 (green) in kidney tissues from the four experimental groups at 6, 24, and 48 h. A, C, E) Representative images at 6 h (**A**), 24 h (**C**), and 48 h (**E**). B, D, F) Corresponding colocalization index based on Pearson’s correlation coefficient at 6 h (**B**), 24 h (**D**), and 48 h (**F**). Mice were divided into four groups: Normal Control; Sepsis (induced by intraperitoneal injection of LPS, 20 mg/kg; Escherichia coli 055:B5); Hypoxia (exposed to 10% O₂ using the ProOx-100 system); and Sepsis-plus-Hypoxia (septic mice placed in the hypoxic chamber immediately after LPS injection). DAPI, 4′,6-Diamidino-2-Phenylindole; LC3, Microtubule-Associated Protein 1 Light Chain 3; TOMM20, Translocase of the Outer Mitochondrial Membrane 20

According to previous study, concurrent elevation of LC3-II with a reduction in translocase of outer mitochondrial membrane 20 (TOMM20) suggests activation of mitophagy, facilitating the clearance of damaged mitochondria and potentially contributing to renal protection [14]. To clarify this, Immunofluorescence co-staining of LC3 and TOMM20 was used to evaluate renal mitophagy at 6-, 24-, and 48-h post-injury (Fig. 5). At 6 h, no significant differences in LC3 and TOMM20 expression or their co-localization were observed among the groups. By 24 h, the Sepsis group began to show increased LC3 and TOMM20 signals with enhanced co-localization, which became more pronounced at 48 h. In contrast, the Sepsis-plus-hypoxia group showed slight increases in LC3 and TOMM20 but less co-localization than the Sepsis group, suggesting a potentially more efficient mitophagic process under combined sepsis and hypoxia conditions.

## Discussion

In our clinical cohort, hypoxemia on ICU admission did not significantly worsen the severity or mortality of SA-AKI, despite the kidney’s well-known vulnerability to hypoxia [15]. To investigatethis clinical observation, we employed an LPS-induced sepsis mouse model. Consistent with the clinical findings, no significant differences were observed between the hypoxia and control groups in renal function, and the addition of hypoxia to sepsis did not further increase SCr, BUN, or histologic tubular injury scores compared to sepsis alone. Therefore, we speculate that a certain degree of hypoxemia may paradoxically confer protective effects during SA-AKI, rather than exacerbating renal injury.

While numerous studies have demonstrated that hypoxia exacerbates renal injury [16]—including our previous work showing that 7% O₂ exposure worsens tubular damage in rats [8]—we propose that the effect of hypoxia is critically dependent on its severity and duration.. In our clinical study, although lower arterial PO₂ levels were initially associated with poorer kidney function and greater AKI severity (Fig. 1C–D), these associations disappeared after multivariable adjustment, indicating that hypoxemia is not an independent determinant of renal injury severity in SA-AKI (Fig. 2B–C). Consistently, Betti et al. found in a large COVID-19 cohort that while lower oxygen saturation at admission was associated with AKI, the link between AKI and mortality was predominantly mediated by worsening respiratory failure, reinforcing the notion that disease severity, rather than isolated hypoxemia, drives outcomes [17]. This phenomenon is physiologically plausible, as the kidney’s exceptionally high basal blood flow (receiving 20–25% of cardiac output) and potent autoregulatory capacity provide a renal oxygen delivery that far exceeds its metabolic demands [18, 19]. We also observed that patients with arterial PO₂ ≥ 100 mmHg exhibited significantly better survival even after adjustment for potential confounders (P < 0.001, Fig. 2D). This dissociation between hypoxemia and renal functional markers, yet persistent association with mortality, suggests that hypoxemia contributes to death primarily through extrapulmonary multiorgan dysfunction rather than direct renal injury [20].

To model hypoxemia in sepsis-associated AKI, we employed an LPS-induced sepsis mouse model combined with a hypoxic chamber to simulate systemic hypoxia. Although the transient cytokine surge in the LPS model does not fully recapitulate the sustained inflammatory response observed in human sepsis, this model reliably reproduces endotoxemia and systemic inflammatory response syndrome, providing a synchronized and quantifiable renal insult [21, 22]. Consistent with our clinical findings (Table 1), LPS injection markedly increased IL-6 and TNF-α expression (Fig. 4A–B). Exposure to 10% inspired oxygen represented a severe hypoxic challenge designed to mimic septic shock and profound hypoxemia. Because arterial PO₂ does not linearly reflect inspired oxygen fraction (FiO₂)—being influenced by alveolar gas exchange, V/Q matching, and metabolic state [23]—a FiO₂ of 10% was selected after careful consideration. Previous studies have shown that moderate hypoxia (e.g., 12% O₂) increases renal susceptibility to secondary insults without structural injury, whereas more severe hypoxia (e.g., 10% O₂) can directly induce renal damage [8, 24]. Moreover, systemic hypoxemia develops gradually as a result of complex physiological adaptations [25]; in our experiment, PO₂ stabilized at 53 mmHg after 48 h (Table 2).

Notably, in our study, mice tolerated the 10% O₂ exposure without overt renal injury, and kidney damage in the sepsis-plus-hypoxia group was even milder than in the sepsis-only group (Fig. 3). Our prior animal research further supports this view: rats exposed to mild hypoxia (10% O₂) demonstrated upregulation of HIF-1α and VEGF, increased renal microvascular density, and no evidence of aggravated tubular injury [8]. Emerging evidence suggests that moderate hypoxia may activate protective pathways without surpassing the threshold for irreversible damage, primarily through stabilization of the HIF signaling pathway, whose activation suppresses apoptosis, downregulates energy-intensive protein synthesis, and promotes metabolic reprogramming to sustain cell survival and maintain homeostasis [26, 27]. This graded response underscores that hypoxia is not uniformly deleterious: while severe hypoxia promotes cellular injury and necrosis, moderate hypoxia may initiate compensatory mechanisms that limit damage and preserve renal function. Understanding the threshold between adaptive and harmful hypoxic responses may help refine oxygen therapy strategies in critically ill patients with SA-AKI.

Mitochondria are central to energy metabolism and are increasingly recognized for their pivotal role in the pathogenesis of SA-AKI. During sepsis, mitochondrial dynamics are significantly disturbed, with a pronounced shift toward excessive fission; notably, such ultrastructural alterations often precede conventional changes in SCr or BUN [28]. In our clinical study, we observed that lactate levels were slightly higher in the ≥ 100 mmHg PO₂ group—a finding that appears to contradict the conventional notion that hypoxia (and resultant anaerobic metabolism) is the major driver of hyperlactatemia (Table 1). Indeed, mounting evidence indicates that septic patients may exhibit normal or even elevated tissue oxygen tensions while demonstrating impaired oxidative phosphorylation and mitochondrial ATP production, leading to compensatory glycolysis and lactate accumulation—findings that point to mitochondrial dysfunction or a catecholamine-driven hypermetabolic state rather than tissue hypoxia per se [29, 30]. Furthermore, in our experimental model we found marked ATP depletion in both Sepsis and Sepsis-plus-hypoxia groups, a finding consistent with previous reports demonstrating that, in the early stages of sepsis, proximal tubular cells may compensate for impaired oxidative phosphorylation by upregulating glycolytic flux to sustain ATP levels [31, 32]. We also observed at 48 h, the Sepsis group showed a relatively higher LC3-II/LC3-I ratio compared to the Sepsis-plus-hypoxia group, which may indicate altered autophagic activity; however, this change could reflect either increased autophagy or autophagy impairment, as well as potential disruption of mitochondrial homeostasis [33].These observations reinforce the notion that PO₂ level may not be the direct driver of renal injury in SA-AKI, and may in fact help explain why the high-PO₂ group in our study still manifested AKI: the renal tubular injury may be predominantly bioenergetic rather than hypoxic in origin.

Although hypoxemia in critically ill patients is often managed with aggressive oxygen supplementation, accumulating evidence suggests that excessive oxygen therapy may paradoxically impair clinical outcomes, including renal function. Recent clinical trials have shown that sustained hyperoxemia (PO₂ > 100 mmHg) within the first 48 h of ICU admission was not associated with improved survival and was linked to longer durations of mechanical ventilation and ICU stay [34]. Excessive oxygen delivery can induce renal vasoconstriction, increase reactive oxygen species production, and trigger inflammatory responses, all of which contribute to tubular apoptosis and structural damage [35, 36]. These data highlight the importance of balancing the risks of hypoxemia with the potential harms of hyperoxia. Our findings support a more nuanced approach, where oxygen therapy should be titrated not simply to normalize peripheral capillary oxygen saturation or PO₂, but guided by overall illness severity, hemodynamics, and organ-specific vulnerability. Importantly, our conclusions should not be interpreted as endorsing permissive severe hypoxemia, which carries well-established risks. Rather, we advocate for judicious oxygen titration that avoids both insufficient oxygen delivery and excessive supplementation—both of which may disrupt the delicate balance of renal oxygen homeostasis.

This study has several limitations. First, in this retrospective study, we included only patients who met the KDIGO criteria for sepsis-associated AKI (Stages 1–3) to specifically assess the effect of hypoxemia in established SA-AKI. Patients without AKI or with mild renal changes below the KDIGO threshold were excluded to maintain cohort homogeneity. Nonetheless, exploring the full continuum from non-AKI to subclinical and overt AKI could provide deeper insight into early renal injury during sepsis. Future prospective studies with sensitive biomarkers are needed to validate these associations and clarify causal relationships. Second, the LPS + 10% O₂ “two-hit” model represents a reductionist approach that reproduces the major inflammatory and hypoxic components of SA-AKI but cannot fully reflect the clinical heterogeneity of human sepsis. Although mild hypoxemia appeared protective, the biological response to hypoxia is dose- and time-dependent. Severe or prolonged hypoxia can induce mitochondrial dysfunction and irreversible tubular injury, and the fixed 10% FiO₂ exposure did not allow assessment of graded hypoxic effects. Third, brief normoxic handling during tissue collection may have influenced hypoxia-sensitive protein measurements, a technical limitation inherent to preclinical hypoxia studies. Moreover, downstream pathways of mitochondrial injury and therapeutic strategies to restore mitochondrial quality control were not explored and warrant further investigation.

## Conclusions

In conclusion, our integrated clinical and experimental analyses suggest that hypoxemia does not necessarily exacerbate renal injury in sepsis-associated AKI, challenging the conventional assumption that higher oxygen delivery is uniformly protective. Through a dual-cohort design, we demonstrate that moderate hypoxemia may engage adaptive responses—particularly those mediated by HIF signaling—that preserve renal function and mitigate mitochondrial stress. These findings underscore the need to reconsider oxygenation strategies in critically ill patients, as excessive oxygen supplementation may disrupt endogenous protective pathways and contribute to secondary injury. Future research should refine oxygenation thresholds that optimize renal outcomes while avoiding the adverse effects of both hypoxemia and hyperoxia, and further elucidate the molecular mechanisms underlying hypoxia-induced renal adaptation.

## Methods

### Inclusion and data collection for patients

Figure 1A provides an overview of both the clinical and animal study designs. We performed a retrospective cohort analysis using data from the Medical Information Mart for Intensive Care IV (MIMIC-IV database, version 1.0), which includes detailed clinical information from 382278ICU admissions at Beth Israel Deaconess Medical Center (Fig. 1B). Access to the database was granted upon completion of the Collaborative Institutional Training Initiative (CITI) program (Author Y.X., Certification No. 43357010). As all data were de-identified, individual informed consent was waived.

Inclusion criteria were as follows: (i) patients aged 18 years or older; (ii) a diagnosis of sepsis confirmed according to Sepsis-3 definitions; and (iii) AKI identified and staged using the Kidney Disease: Improving Global Outcomes (KDIGO) guidelines. Exclusion criteria included: (i) repeated ICU admissions; (ii) ICU length of stay less than 48 h; (iii) incomplete data, particularly missing arterial blood gas or renal function measurements; (iv) presence of chronic kidney disease prior to ICU admission; and (v). patients who received mechanical ventilation, within the first 24 h after ICU admission.

Clinical data were extracted from the MIMIC database, including demographic variables (age, gender, ethnicity), clinical information (first care unit and severity of illness assessed by the SOFA, APSIII, and Charlson Comorbidity Index scores), oxygen metabolism variables (PO₂, partial pressure of carbon dioxide-PCO₂, lactate), inflammatory markers (WBC, NLR), renal injury indicators (BUN, SCr), intervention variables (mechanical ventilation and use of vasoactive agents), and outcomes (in-hospital mortality and length of hospital stay). Age, gender, ethnicity, first care unit, and severity-of-illness scores were obtained at ICU admission. Oxygen metabolism, inflammatory, and renal injury variables were recorded as the highest values within the first 24 h after ICU admission. Intervention variables were defined as the use of mechanical ventilation or vasoactive agents within the first 24 h of ICU stay.

Based on the highest PO₂ within the first 24 h after admission, patients were categorized into four groups: < 60 mmHg, ≥ 60 and < 80 mmHg, ≥ 80 and < 100 mmHg, and ≥ 100 mmHg. Non-parametric tests were used to compare renal function indicators among the four groups, as well as for pairwise comparisons, to evaluate differences in renal function. To further clarify the association between arterial PO₂ and renal injury markers (BUN and SCr), multiple linear regression analyses were performed. Potential confounding factors were controlled for, including age, gender, ethnicity, SOFA score, APSIII score, Charlson comorbidity index, WBC NLR, and vasoactive medication use. In addition, K-M survival analyses were conducted to investigate the relationship between arterial PO₂ levels and patient outcomes.

### Construction of a murine sepsis model

All animal procedures were approved by the Ethics Committee of Xinhua Hospital, Shanghai Jiao Tong University School of Medicine (XHEC-F-2024–004) and conducted in accordance with institutional guidelines. Male C57BL/6 J mice (20–25 g, 8–10 weeks old) were randomly assigned to four groups (n = 6 each): Normal control; Sepsis, induced by intraperitoneal injection of 20 mg/kg (LPS, Escherichia coli 055:B5; Sigma-Aldrich, USA) [37]; Hypoxia, exposed to a 10% O₂ environment (ProOx-100 system, Tawain Intelligent Technology, Shanghai); Sepsis‑plus‑hypoxia group, in which septic mice were placed in the hypoxic chamber after LPS injection. The 10% hypoxic environment was selected based on our previous studies, in which we observed renal self-repair mechanisms without significant kidney injury at this oxygen concentration [8, 38].

Survival and physiological responses were monitored throughout. Mice were euthanized at 6, 24, and 48 h, and samples were collected. Arterial blood gas analysis was performed using ABL800 FLEX (Radiometer), and SCr was measured via automated biochemical analyzer (Hitachi, Tokyo, Japan). One kidney was snap-frozen in liquid nitrogen for WB; the other was fixed in 4% paraformaldehyde for histological and immunofluorescent analysis.

### Histology and tubular damage score

Kidneys were fixed in 4% paraformaldehyde for 24 h, embedded in paraffin, and sectioned at the maximal transverse plane. Haematoxylin and eosin (H&E) staining was performed. Blinded histological evaluation was conducted by two independent investigators. Ten random cortical fields per mouse were assessed. Tubular injury was scored as: 0 (none), 1 (< 25%), 2 (25–50%), 3 (51–75%), and 4 (> 75%) of tubules showing damage [39].

### Adenosine triphosphate quantification

Tissue lysates (200 µL per 20 mg tissue) were prepared, and supernatants analyzed using an Enhanced Adenosine Triphosphate Assay Kit (S0027, Beyotime, China). ATP levels were normalized to total protein content (µmol/mg protein), quantified using a microplate reader.

### Western blot analysis

Frozen kidney samples were lysed in 500 µL buffer, centrifuged at 4 °C, and stored at − 80 °C. Protein concentrations were determined via the Bradford assay. Blots were probed with anti-cytosolic microtubule-associated protein 1 light chain 3, anti-LC3-I (1:1000, Proteintech, USA) and anti-β-actin (1:1000, Sigma, USA).

### Immunofluorescence and colocalization analysis

Paraffin sections were deparaffinized, blocked, and antigen-retrieved, followed by incubation with primary antibodies: LC3 (1:200, Proteintech) and translocase of outer mitochondrial membrane 20, TOMM20 (1:5000, Servicebio, China) at 4 °C overnight. Nuclei were counterstained with 4′,6-diamidino-2-phenylindole, DAPI (Servicebio). Images were acquired using a Nikon fluorescence microscope and analyzed with 3DHISTECH software.

### Statistical analysis

Normality was assessed using the Shapiro–Wilk test. Parametric data were expressed as mean ± standard deviation (SD) and compared among groups using one-way analysis of variance (ANOVA). Non-parametric data were presented as median (P25, P75) and compared using the Kruskal–Wallis test, followed by pairwise comparisons when appropriate. Categorical variables were analyzed via chi-square or Fisher’s exact test. Image J (JACoP plugin) was used for colocalization quantification. All analyses were conducted in SPSS version 25 (IBM, USA), with P < 0.05 considered statistically significant.

## Supplementary Information

Supplementary Material 1. Figure S1. Representative kidney histology at 6, 24, and 48 h, with hematoxylin and eosin staining.

Supplementary Material 2. Figure S2. Relative density oLC3-II/LC3-I ratio, detected by WB.

## Data Availability

The data used in this study were obtained from the MIMIC database, a publicly available and de-identified critical care database maintained by the Massachusetts Institute of Technology. The datasets used and/or analyzed during the current study are available from the corresponding author on reasonable request.

## Abbreviations

AKI: Acute Kidney Injury
ANOVA: Analysis of Variance
APSIII: Acute Physiology Score III
ATP: Adenosine Triphosphate
BUN: Blood Urea Nitrogen
DAPI: 4′,6-Diamidino-2-Phenylindole
FiO₂: Fraction of Inspired Oxygen
H&E: Hematoxylin and Eosin
HIF-1α: Hypoxia-Inducible Factor 1 Alpha
ICU: Intensive Care Unit
IL-6: Interleukin-6
KDIGO: Kidney Disease: Improving Global Outcomes
K-M: Kaplan–Meier
LC3: Microtubule-Associated Protein 1 Light Chain 3
LPS: Lipopolysaccharide
MIMIC-IV: Medical Information Mart for Intensive Care IV
NLR: Neutrophil-to-Lymphocyte Ratio
PCO₂: Partial Pressure of Carbon Dioxide
PO₂: Partial Pressure of Oxygen
SA-AKI: Sepsis-Associated Acute Kidney Injury
SCr: Serum Creatinine
SD: Standard Deviation
SOFA: Sequential Organ Failure Assessment
TNF-α: Tumor Necrosis Factor Alpha
TOMM20: Translocase of Outer Mitochondrial Membrane 20
VEGF: Vascular Endothelial Growth Factor
WB: Western Blot
WBC: White Blood Cell
