# Supplementary figures and images for: Does hypoxemia aggravate sepsis-associated acute kidney injury? Integrated clinical and experimental evidence
Source: Intensive Care Med Exp. 2025 Dec 21;13:134. doi: 10.1186/s40635-025-00840-x (PMC12718267; doi:10.1186/s40635-025-00840-x)

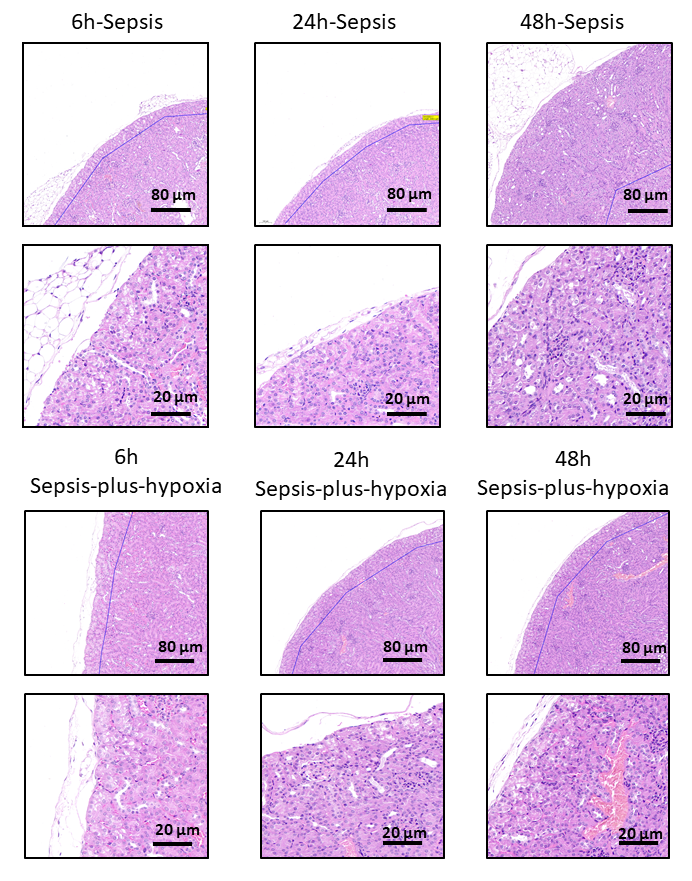

Supplement: Supplementary file 1 — Supplementary Material 1. Figure S1. Representative kidney histology at 6, 24, and 48 h, with hematoxylin and eosin staining. [file 40635_2025_840_MOESM1_ESM.tif]

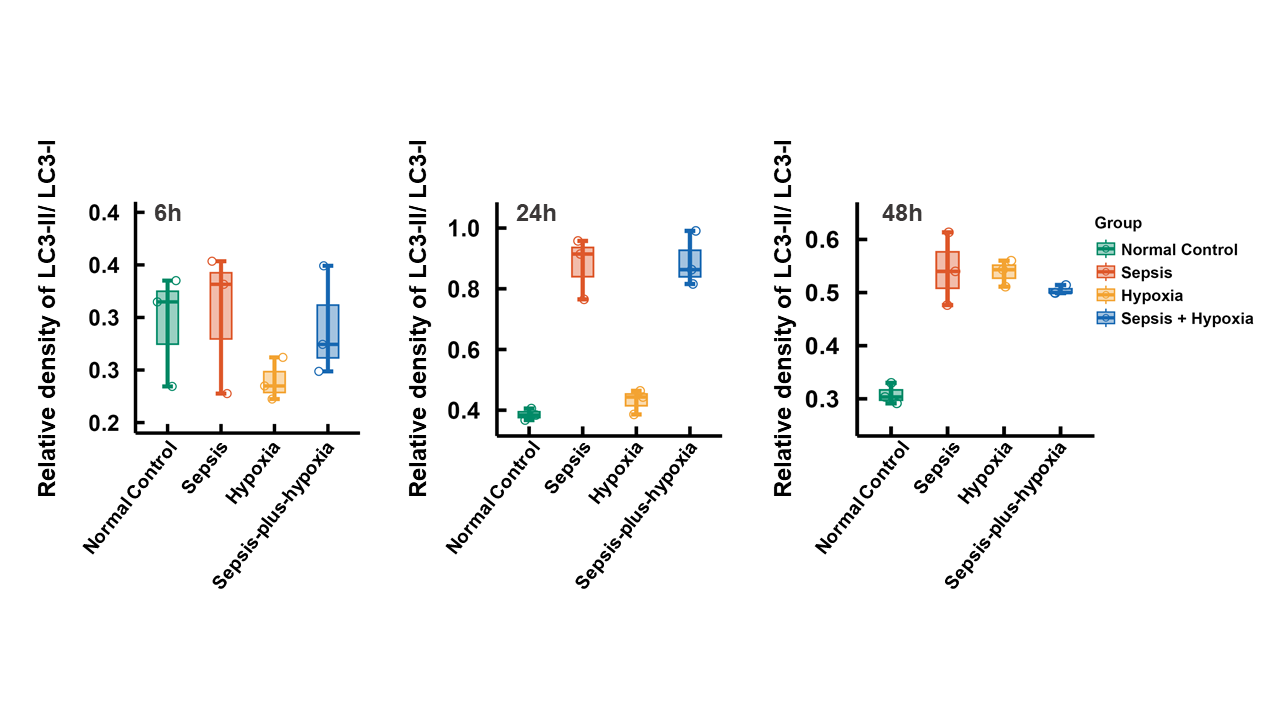

Supplement: Supplementary file 2 — Supplementary Material 2. Figure S2. Relative density oLC3-II/LC3-I ratio, detected by WB. [file 40635_2025_840_MOESM2_ESM.tif]
